# Prosocial skills may be necessary for better peer functioning in children with symptoms of disruptive behavior disorders

**DOI:** 10.7717/peerj.487

**Published:** 2014-07-15

**Authors:** Brendan F. Andrade, Dillon T. Browne, Rosemary Tannock

**Affiliations:** 1 Centre for Addiction and Mental Health, Toronto, ON, Canada; 2 Department of Psychiatry, University of Toronto, Toronto, ON, Canada; 3 Ontario Institutes for Studies in Education, University of Toronto, Toronto, ON, Canada; 4 Hospital for Sick Children, Toronto, ON, Canada

**Keywords:** Conduct Disorder, Oppositional Defiant Disorder, Prosocial skills, Moderation, Mediation, Peer problems

## Abstract

Children with disruptive behavior disorders experience substantial social challenges; however, the factors that account for (i.e., mediate), or influence (i.e., moderate), peer problems are not well understood. This study tested whether symptoms of Oppositional Defiant Disorder and Conduct Disorder were associated with peer impairment and whether prosocial skills mediated or moderated these associations. Teacher ratings were gathered for 149 children (Mage = 9.09, *SD* = 1.71, 26% female) referred for behavioral concerns to an urban child psychiatry clinic. Path-analytic linear regressions testing mediation and moderation effects showed that prosocial skills significantly moderated the negative effects of symptoms of Conduct Disorder on peer impairment. Children showed less peer impairment only when they had relatively few conduct symptoms and high prosocial skills. Measurement of prosocial skills, in addition to conduct problems, may best capture factors which contribute to peer problems of children with disruptive behaviors.

## Introduction

Children with disruptive behavior disorders such as Oppositional Defiant Disorder (ODD) and Conduct Disorder (CD) experience substantial challenges in multiple life domains (Pardini & Fite, 2010; Rowe et al., 2010). Paramount amongst these challenges are marked problems with peers, which include peer rejection, peer neglect, and social-behavior and social-skills difficulties (Dodge et al., 2003; Dodge et al., 1986; Hoza et al., 2005b; Landau, Milich & Diener, 1998). To help reduce the negative social and behavioral impacts of psychopathology it is necessary to understand the factors that contribute most to peer problems in children with disruptive behaviors.

Peer relationship difficulties have emerged as a salient and important predictor of mental health and behavioral adjustment (Milich & Landau, 1984; Miller-Johnson et al., 2002; Ollendick et al., 1992; Silver et al., 2010). Children with impaired peer relationships are at elevated risk of compounding problems in multiple domains of their life (Dodge & Pettit, 2003; Ollendick et al., 1992). In contrast, adaptive peer relationships appear to buffer children, possibly through the development of positive social connections (Criss et al., 2002). Understanding the factors that most closely contribute to problematic peer functioning is critical to help prevent or mitigate the many short and long-term negative social and behavioral outcomes experienced by children with disruptive behavior.

Although associations between ODD, CD and problematic social functioning are somewhat established, many gaps in knowledge still exist. First, much previous research has investigated social functioning in children diagnosed with ODD or CD (Burke, Loeber & Birmaher, 2002; Pardini & Fite, 2010). Although informative and important, these studies omit a large number of children with symptoms of disruptive behavior disorders who are diagnostically subthreshold. Although not meeting DSM-IV criteria for ODD or CD, subthreshold children may also experience marked social difficulties (Drabick & Gadow, 2012; Hofvander et al., 2009). Categorization based on diagnostic cutoffs neglects relationships between the *degree* of symptoms and social impairment. Because of the severity and salience of symptoms comprising ODD and CD, it is possible that even a few symptoms may contribute to peer impairment (Drabick & Gadow, 2012). Investigation of the association between ODD and CD symptom dimensions with peer impairment is necessary to augment current understanding of symptom-impairment relationships (Lahey & Waldman, 2012).

Second, existing research has often neglected inclusion of other specific aspects of social behavior, which may influence *or* account for some of the association between symptoms of ODD and CD with peer problems. Studies that describe social behaviors that are positive, or prosocial, have received increased attention because of their important relationship with social functioning (Coie & Dodge, 1988; Eisenberg et al., 2001; Nelson & Crick, 1999; Seligman & Csikszentmihalyi, 2000). Specifically, prosocial skills in childhood are associated with higher peer status, less rejection and more adaptive social outcomes (Coie, Dodge & Kupersmidt, 1990; Crick, 1996). Children who exhibit strong prosocial skills may be capable of managing social challenges and most socially competent. Prosocial skills, when implemented effectively, may influence and somewhat counter the negative impacts of disruptive behaviors. This assertion is consistent with research that shows children who demonstrate aggressive behavior and prosocial skills have high social status (Blake, Kim & Lease, 2011; Rodkin et al., 2000). As such, prosocial skills may lessen, or moderate, the negative impact of symptoms of ODD and CD on peer impairment (Carson, 2013). Alternatively, prosocial skills may be intimately connected and of salient importance to peers (Eisenberg et al., 2001; Greener & Crick, 1999). It is possible that prosocial skills are necessary for appropriate peer functioning (Andrade & Tannock, 2013); however, children with symptoms of ODD or CD may not be exposed to situations or models that help develop their prosocial skills (Romano et al., 2005). Children with elevated ODD or CD symptoms may not have adequate knowledge of prosocial skills or how to implement these skills (Dodge & Pettit, 2003; Perren et al., 2007). Prosocial skills knowledge or implementation deficits may contribute to peer problems in children with disruptive behavior (Crick, 1996; Perren et al., 2007). As such, prosocial skills may partly account for, or mediate, the association between symptoms of ODD and CD with peer impairment. That is, prosocial skills may be an important link in the causal chain connecting symptoms of ODD and CD with peer impairment (Criss et al., 2002; Doyle et al., 2003; Mikami & Hinshaw, 2003). Although each plausible, these two related and important assertions are in need of investigation.

Finally, although empirically supported psychosocial treatments exist for children with disruptive behavior disorders, few effective treatments specifically target social impairment (Hoza et al., 2005a; La Greca, Silverman & Lochman, 2009; Mikami et al., 2013). Current well-established treatments primarily target development of children’s emotional, behavioral, and problem-solving skills (Kazdin, Siegel & Bass, 1992; Lochman & Wells, 2004; Webster-Stratton & Reid, 2003). Limited research shows positive effects of interventions primarily geared to augment social behavior (Mikami et al., 2011; Mikami et al., 2013). An increased understanding of the direct, indirect and contingent associations of ODD and CD symptoms and social impairment will inform the development of components to augment intervention effectiveness.

The overall objective of the present study was to test whether *symptom dimensions* of ODD and CD were related to social impairment and whether these associations were moderated *or* mediated by prosocial skills. The following hypotheses were proposed. First, elevated symptoms of ODD and CD would each be associated with greater peer impairment. Second, prosocial skills would lessen the negative impact of symptoms of ODD and CD on peer impairment, such that higher levels of prosocial skills would be associated with less severe peer impairment for children with many symptoms of ODD and CD. Third, prosocial skills would partially account for the association between symptoms of ODD and CD with peer impairment.

## Method

### Participants and procedures

Participant data was gathered from an existing database of 149 participants in a larger-scale research project on attention and working memory being conducted at a Neuropsychiatry clinic in an urban pediatric hospital in Toronto, Canada. Participants were referred to the clinic because of behavioral concerns and query of Attention Deficit Hyperactivity Disorder (ADHD). Participants were not recruited for the issues under investigation in the present study. All participants had a Full scale IQ (FSIQ) of 80 or greater, did not meet criteria for Learning Disability, Autism, Psychosis or show evidence of neurological dysfunction. Participant demographics and descriptives are displayed in Table 1.

**Table 1:** Descriptive statistics and variable intercorrelations.

	IA	HI	ODD	CD	PI	Pro	Female	*M*	*SD*
Age	−0.02	−0.14	.18^*^	−0.01	0.04	−.17^*^	0.02	9.09	1.71
Inattention (IA)		.41^**^	.17^*^	0.11	0.07	0.13	−0.01	4.88	1.98
Hyperactivity (HI)			.26^**^	.23^**^	.42^**^	−0.07	−0.15	3.77	2.20
Opposition (ODD)				.55^**^	.50^**^	−.24^**^	−.17^*^	1.47	1.77
Conduct (CD)					.29^**^	−0.13	−0.15	0.29	0.71
Peer impairment (PI)						−.17^*^	0.03	1.63	1.01
Prosociality (Pro)							.17^*^	1.51	0.42

**Notes.**

* *p* < .05.

** *p* < .01.

Comprehensive assessments done with children for clinical diagnostic purposes involved DSM-IV based semi-structured parent and teacher interview and completion of questionnaires. The Parent Interview of Child Symptoms (PICS) (Ickowicz et al., 2006) and Teacher Telephone Interview-IV (TTI-IV) (Tannock et al., 2002) are semi-structured interviews which were conducted by the Psychiatrist, Psychologist or Social Worker, to assess parent and teacher perceptions of the child in a variety of family or educational contexts.

In addition, parents and teachers completed a number of standardized rating scales (e.g., Connors Parent Rating Scale–R, Connors Teacher Rating Scale–R) to provide complementary diagnostic information used for clinical purposes. All cases were reviewed by a clinical team comprised of a Psychiatrist, Psychologist, and Social Worker, who confirmed or ruled out diagnoses. Detailed descriptions of assessment procedures used have been reported previously (Bedard et al., 2003). Ethical approval to conduct the project was provided by the Hospital for Sick Children Research Ethics Board (file number 1000004481) and parental consent to undertake additional research with the data was obtained at the time of initial data collection.

### Measures

#### Teacher telephone interview-IV (TTI-IV)

Symptoms of ODD, CD, Inattention, and Hyperactivity-Impulsivity used in this study were assessed using the TTI-IV (Tannock et al., 2002), a semi-structured interview conducted over the telephone to assess the teacher’s perceptions of the child in a variety of educational contexts. These contexts include structured activities, such as doing classroom seatwork, and unstructured activities, such as transitions and play. Behavioral descriptions provided by the teacher are probed if necessary, and scored based on DSM-IV criteria. The clinician determined the presence or absence and severity of symptoms based on description provided by the teacher. In this study internal consistency of the ODD and CD scales were 0.82 and 0.65 respectively. Note that these reliabilities are Kuder-Richardson (KR-20) coefficients, as items (symptoms) are dichotomous.

#### Strength and difficulties questionnaire (SDQ)

The SDQ is a brief screening questionnaire that can be administered to both teachers and parents using parallel forms (Bourdon et al., 2005; Goodman et al., 2003). The questionnaire enquires about 25 attributes that are evenly divided among five behavioral dimensions (5 items per behavioral dimension); prosocial skills, emotional symptoms, conduct problems, hyperactivity-inattention, and peer problems. Subscales do not overlap, and each produces a total score. The teacher-reported prosocial skills scale was used in this study. This scale includes five questions: “Considerate of other people’s feelings”, “Shares readily with other children (treats, toys, pencils, etc.)”, “Often volunteers to help others”, “Kind to younger children” and “Often offers to help others”. Each item is rated on a 3-point Likert scale ranging from Not True, Somewhat True to Certainly True. The scale shows strong reliability, internal consistency and validity in past studies (Bourdon et al., 2005; Stone et al., 2010). In this study internal consistency of the prosocial skills scale was 0.86.

### Peer impairment rating

The peer impairment rating was included as a supplement to the SDQ to assess impairment in a number of school and social domains. Specifically, the peer impairment scale is a 6-point Likert rating (0 = “not at all” and 5 = “a great deal”) used to assess teachers’ views of the negative impact of the child’s behavioral difficulties on peer relationships (i.e., Do the child’s difficulties interfere with their peer relationships?). The validity and reliability of teacher ratings of peer behavior have been supported in a number of studies (Andrade, Waschbusch & King, 2005; Coie & Dodge, 1988; Ledingham et al., 1982).

### Analysis

Path-analytic linear regression using maximum likelihood estimation in MPlus Version 7.0 was used to determine if prosocial skills either mediated or moderated the relationship between disruptive behavior (ODD or CD symptoms) and peer impairment. Given that both domains of disruptive behavior are highly collinear but diagnostically distinct, two models were run and evaluated separately. In each model, peer impairment was regressed onto prosocial skills, the disruptive behavior in question (ODD or CD symptoms), and an interaction term (the product of either ODD or CD symptoms and prosocial skills). Additionally, the prosocial skills variable was regressed onto the disruptive behavior in question. This was done in order to permit the possibility of an indirect effect of the disruptive behavior on peer impairment via prosociality. The standard errors of the indirect effects were evaluated using the bootstrapping procedure (1,000 draws). All analyses controlled for age and gender. Additionally, due to the referred nature of the sample, models controlled for symptoms of inattention and hyperactivity-impulsivity. Model fit was evaluated using the chi-square test, Tucker-Lewis Index Index (TLI), Root Mean Square Error of Approximation (RMSEA), and Standardized Root Mean Square Residual (SRMR). Regression diagnostics were conducted and model assumptions were tested (e.g., collinearity, homoscedasticity, normality of residuals). In accordance with (Graham, 2009), missing data was explored and described using Little’s Test (Little, 1988). Missingness was accounted for using Estimation Maximization in SPSS 20 (descriptive statistics and correlations) and Full Information Maximum Likelihood in MPlus 7.0 (model fitting).

## Results

### Missing data and descriptive statistics

Of the 149 participants there was some missing data that ranged from no missing (gender variable) to 12% (peer problems variable). The main reason for missingness was an inability to obtain complete assessment materials from some teachers. To assess the pattern of missingness, Little’s Missing Completely at Random test (MCAR) was conducted. This test was not significant, *χ*^2^(35) = 28.21, *p* = .785, indicating that the MCAR assumption was not violated. Thus, it is reasonable to assume that the missing values represent a random subset of values and that there is no systematic bias in patterns of missingness. Descriptive statistics are presented in Table 1.

#### Is the association between CD symptoms and peer impairment mediated and/or moderated by prosocial skills?

**Figure 1:**
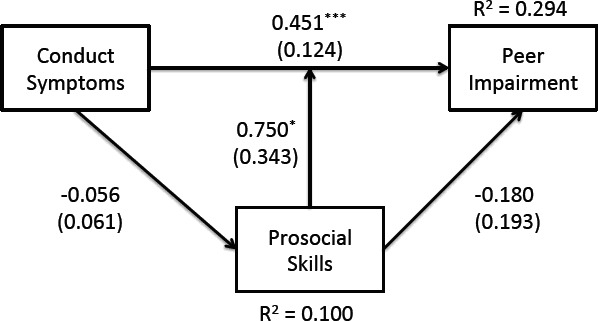
Pathway regression output depicting that the relationship between Conduct Disorder symptoms and peer impairment is moderated (not mediated) by prosocial skills.

**Figure 2:**
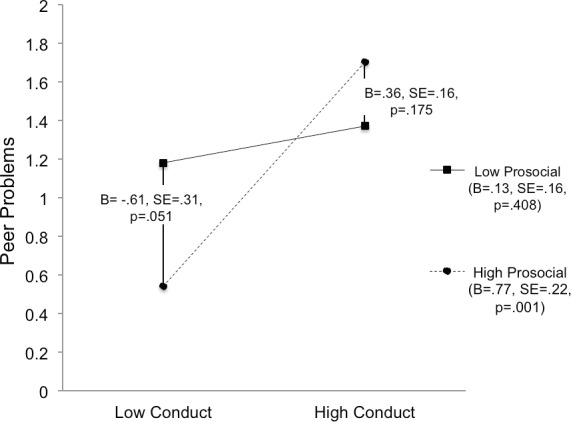
Plot of the Conduct Disorder symptoms and prosocial skills interaction. The children with the least peer impairment have low CD symptoms and high prosocial skills.

The model for CD symptoms is presented in Fig. 1. Fit indices suggest that the model fit the data well, *χ*^2^(1) = .701, *p* = .402, TLI > .99, RMSEA < .01, SRMR = .010. There was a significant main effect of CD symptoms on peer impairment, whereby children with higher CD symptoms had significantly more peer impairment. There was not a significant main effect of prosocial skills on peer impairment. However, there was a significant CD-by-prosocial interaction, suggesting that the association between symptoms of CD and peer impairment varied as a function of an individual’s level of prosocial skills (i.e., evidence of moderation). This interaction is plotted in Fig. 2.

Simple slopes analysis was conducted in order to better understand the interaction (Holmbeck, 2002). First, the effect of CD symptoms at high (i.e., more skills) and low levels of prosocial skills was examined (i.e., the slopes of the two lines in Fig. 2). At high levels of prosocial skills (+1 standard deviation), more CD symptoms are associated with significantly more peer impairment (*B* = .77, *SE* = .22, *p* = .001). However, at low levels of prosocial skills (−1 standard deviation), more CD symptoms are not associated with an increase in peer impairment (*B* = .13, *SE* = .16, *p* = .408). As such, children with few prosocial skills showed elevated peer impairment at low and high levels of CD. Next, the association between prosocial skills and peer impairment at high and low levels of CD symptoms was examined (i.e., the difference in the end-points of the two different lines in Fig. 2). At high levels of CD symptoms, prosocial skills were not significantly associated with peer impairment (*B* = .36, *SE* = .26, *p* = .175). However, at low levels of CD symptoms, individuals who had more prosocial skills had significantly lower ratings of peer impairment (*B* = −.61, *SE* = .31, *p* = .051). To summarize, the only children with relatively little peer impairment were those who had few CD symptoms *plus* high prosocial skills.

There was no evidence of prosocial skills mediating CD symptoms. That is, the direct effects of CD symptoms on prosocial skills, and of prosocial skills on peer impairment, were non-significant (i.e., no reason to test the indirect effect). Tests of the control variables revealed that children with higher levels of hyperactivity had significantly greater peer impairment (*B* = .18, *SE* = .04, *p* < .001). Also, after adjusting for all other factors, females had higher levels of peer impairment (*B* = .40, *SE* = .20, *p* = .039). Lastly, older children had lower levels of prosocial skills (*B* = −.05, *SE* = .02, *p* = .008), and children with higher levels of inattention had lower prosocial skills (*B* = −.04, *SE* = .02, *p* = .036).

#### Is the association between ODD symptoms and peer impairment mediated or moderated by prosocial skills?

**Figure 3:**
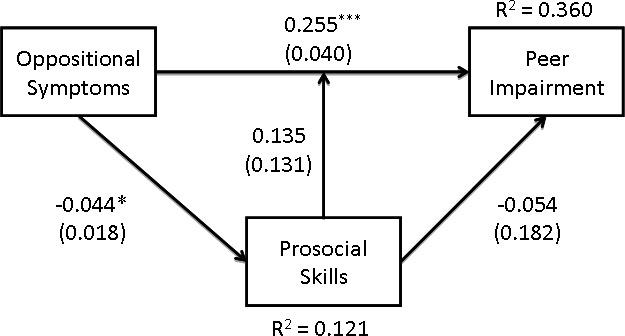
The association between oppositional symptoms and peer impairment is neither mediated nor moderated by prosocial skills.

The model for ODD symptoms is presented in Fig. 3. The TLI and the RMSEA suggested that this model did not fit the data particularly well, *χ*^2^(1) = 2.48, *p* = .116, TLI = .67, RMSEA = .10, SRMR = .02. Although there was a significant main effect of symptoms of ODD on both peer impairment and prosocial skills, there was no indication of moderation. Also, given that there was no significant direct relation between prosocial skills and peer impairment, any possibility of mediation is similarly ruled out. Thus, the effect of symptoms of ODD on peer impairment appeared to be independent of prosocial skills, both in terms of moderation and mediation. Neither the effects of age or gender were significant in this model.

## Discussion

This study tested whether symptoms of ODD and CD were associated with peer impairment and whether these associations were moderated or mediated by prosocial skills in a sample of children referred to an urban children’s mental health clinic. We hypothesized that symptoms of disruptive behavior would be associated with greater peer impairment and prosocial skills would moderate and partially mediate the relationships. Results showed that symptoms of ODD and CD were each significantly associated with peer impairment. Contrary to expectation, prosocial skills did not partially account for the association between symptoms of ODD or CD with social impairment. However, the relationship between CD symptoms and peer impairment varied as a function of prosocial skills, such that children with less severe CD showed relatively less social impairment, but *only if* they had high prosocial skills.

Findings show that prosocial skills have an important influence on the association between symptoms of CD and peer functioning. This finding is salient given that children who show early symptoms of conduct problems are at much greater risk for antisocial behavior, substance abuse, school drop-out, relationship and mental health problems in adolescence and adulthood (Burke, Loeber & Birmaher, 2002; McMahon, Wells & Kotler, 2006; Rowe et al., 2010). However, contrary to our hypotheses, prosocial skills did not show a protective or buffering effect at relatively high levels of Conduct Disorder. Rather, the only children in the study who were doing relatively better in terms of peer functioning had low levels of conduct problems and high prosocial skills. Researchers have focused on the elucidation of factors that lessen the negative impacts of symptoms of CD, thereby informing intervention approaches to mitigate risk of these negative outcomes. The current study suggests that lower levels of conduct problems *and* higher prosocial skills may be necessary for better peer functioning. This finding has implications for intervention. For children with elevated levels of conduct problems, programs that mainly target reduction in conduct problem behavior without adequately developing prosocial skills may be insufficient to impact peer functioning. Development of prosocial skills, in multicomponent programs that also target reduction in conduct problems, may be important in order to impart change in social outcomes for children with conduct problems. A growing body of research highlights the importance of prosocial skills for the development of appropriate peer relationships (Crick, 1996; Eisenberg et al., 2001; Mayeux & Cillessen, 2003; Parkhurst & Asher, 1992; Perry, Perry & Rasmussen, 1986). Further research to illuminate the shared contribution of prosocial skills and conduct problems to peer functioning would be beneficial.

It is important to note that *symptoms* of ODD and CD when treated dimensionally were each associated with greater peer impairment. Although ODD showed a stronger relationship with peer impairment compared to CD, both were statistically significant. This finding points to the important relationship between symptom severity and social impairment, extending findings from diagnosed and undiagnosed groups of children (Drabick & Gadow, 2012; Pope, Bierman & Mumma, 1991). Although referred for clinical assessment, many children in the present study did not receive diagnoses of ODD or CD; however most showed symptomatology associated with social impairment. Results highlight the important influence of symptom dimensions on social functioning—a finding that is somewhat intuitive given the salience of individual symptoms of ODD and CD and logical links to social problems. For example, symptoms such as “often bullies, threatens or intimidates others” or “often initiates physical fights” may be sufficiently salient to cause peer problems regardless of other symptoms. Although beyond the scope of this study, future studies to determine the specific association between individual symptoms, or clusters of symptoms, with specific social problems would be informative.

Further, correlational findings from this study show that increased prosocial skills were associated with fewer symptoms of ODD and CD and less peer impairment. Although these findings should be considered preliminary, further research to determine the mechanisms by which prosocial skills influence the severity of disruptive behavior, and which prosocial skills are most closely linked to adaptive peer functioning, is important to more clearly specify potential avenues for intervention.

Contrary to hypotheses, no significant mediation effects were found. In this sample of children, prosocial skills were not part of the causal chain linking symptoms of disruptive behavior and peer impairment. This null finding is important and may indicate that symptoms of disruptive behavior and prosocial skills are not part of the same mechanism leading to peer problems. However, findings did show that prosocial skills lessened the negative influence of less severe CD symptoms on social impairment. As such, conduct problems and prosocial skills may act via different-but-complementary mechanisms to impact peer functioning. This finding highlights the value of considering positive social behaviors, in addition to symptoms of disruptive behavior, when determining factors that convey risk to the development of social problems.

Of note, although fewer prosocial skills were significantly associated with greater symptoms of ODD, prosocial skills did not significantly mediate or moderate the association between symptoms of ODD and peer impairment. Symptoms of ODD may be experienced differently by peers than CD (Burke, Loeber & Birmaher, 2002). Symptoms of ODD may be troubling to peers in a way that cannot be compensated by prosocial skills (Drabick & Gadow, 2012; Frankel & Feinberg, 2002; Pardini & Fite, 2010). For example, symptoms such as “often loses temper”, “often deliberately annoys people”, “often blames others for his or her mistakes or misbehavior” are somewhat incompatible with prosocial skills and may override any positive influence of prosocial skills. For children with symptoms of ODD, reduction of these problematic behaviors may be of primary importance to promote adaptive peer functioning. Although plausible, these assertions are in need of further systematic examination.

### Study limitations

Although findings provide novel information, some limitations should be considered. First, because the study design is cross-sectional, causal mechanisms cannot be inferred. However, results clearly describe direct and indirect relationships between symptoms of ODD, CD, and prosocial skills that may provide important foundational information for a larger scale longitudinal study to test symptom and impairment relationships. Moreover, this study purposely investigated the contributions of symptoms of ODD and CD to peer impairment; however, other psychological, behavioral or social factors were not investigated. These additional factors may have an important influence on peer functioning and should be investigated using more comprehensive statistical models and larger data sets. Second, sex differences were controlled in the analysis and were not a primary variable tested due to the small proportion of females in the present sample. However, girls showed higher levels of peer impairment after ODD, CD and prosocial skills were taken into account. We speculate that this finding may be due to relational aggression and other behavioral characteristics more closely attributed to girls but not measured in this study. Findings may be different if examined in populations of girls, given developmentally stronger verbal abilities and higher rates of relational aggression than boys (Crick & Werner, 1998). Third, the use of teacher report of ODD and CD symptoms and peer functioning has been used in numerous past studies to quantify children’s school-based behaviors (Coie & Dodge, 1988; Drabick & Gadow, 2012; Waschbusch, Sparkes & Northern Partners In Action for Children and Youth, 2003). Although valuable, teacher reports could be augmented in larger scale studies with direct observation, peer sociometrics and other naturalistic measures.

### Research implications

The present study highlights the unique and shared impacts of symptoms of disruptive behavior and prosocial skills on children’s social functioning. Of particular importance is the emphasis on the contribution of specific symptom categories to disrupted childhood relationships. We hope that this approach to analyses will be used in more research to describe aspects of disruptive and prosocial skills that are associated with or predict childhood social impairment and adaptive social behavior. For example, further testing the association between specific symptoms, or symptom clusters of ODD and CD, and specific subtypes of prosocial skills and social impairment will lead to a better understanding of the direct connection between symptoms and real world functioning. Additionally, more clearly understanding which specific symptoms of disruptive behavior confer the most risk of social impairment would be beneficial.

Research and development of targeted and effective individualized and group-based treatments for social problems would benefit from a targeted skills building approach. Treatments that emphasize the joint development of prosocial skills and reduction of conduct problems may have the most positive social impacts. Addressing both disruptive and adaptive behavior in a multicomponent intervention may be a synergistic and effective way to best reduce the social distress experienced by many children with disruptive behavior.

### Clinical implications

Prosocial skills in children with conduct problems may have an important and understudied application to intervention. Reducing symptoms of CD may only have a positive impact on social behavior with *appropriate* facilitation of prosocial behavioral skills. Development of positive behavioral skills may uniquely lessen social impairment and increase the quality of relationships. In fact past research has demonstrated the unique contribution of prosocial skills to adaptive child development and the positive impact of prosocial skills on peer relationships (Crick, 1996; Denham, 1986; Eisenberg et al., 2001). Interventions that specifically target prosocial skills have demonstrated short and longer-term positive impacts on social relationships; however, these are few (Bowers et al., 2000; Cashwell, Skinner & Smith, 2001). Additional investigation of specific interventions that target prosocial skills and their impact on peer functioning are much needed.

From an assessment perspective, results provide evidence of the importance of prosocial skills, along with disruptive behavior, when determining a child’s level of functioning. In this study, prosocial skills and symptoms of disruptive behavior influenced degree of peer impairment; therefore, a thorough assessment of a child’s challenges and strengths should likely include typically administered measures of disruptive behavior, along with complementary measures of prosocial skills. This form of assessment would provide a more appropriate reflection of the child’s functioning and closely inform approaches to treatment planning and strategies for intervention.
